# Are we saying it right? Communication strategies for fighting vaccine hesitancy

**DOI:** 10.3389/fpubh.2023.1323394

**Published:** 2024-01-05

**Authors:** Antonio Di Lorenzo, Pasquale Stefanizzi, Silvio Tafuri

**Affiliations:** ^1^Department of Interdisciplinary Medicine, School of Medicine, University of Bari Aldo Moro, Bari, Italy; ^2^Department of Interdisciplinary Medicine, University of Bari Aldo Moro, Bari, Italy

**Keywords:** vaccine advocacy, anti-vaccination arguments, vaccine literacy, healthcare workers training, vaccination coverage

## Abstract

Vaccine hesitancy is a multi-faceted phenomenon, deeply rooted in cultural, socioeconomic and personal background. Communication is deemed fundamental in fighting vaccine hesitancy. Medical communication should be accessible, relying both on an emotional approach and accurate information. Trained professionals should curate communication with the public.

## Introduction

According to current definition, vaccine hesitancy (VH) consists in “delay in acceptance or refusal of vaccination despite availability of vaccination services” (1). It has been identified by the World Health Organization as one of the 10 most serious threats to global health since it hinders vaccination efforts, thus creating vulnerable niches of individuals in which infection diseases’ outbreaks might occur (2). The loss of community immunity due to suboptimal vaccination coverage also increases the risk of vaccine preventable diseases and their complications for vulnerable subjects who failed to respond to vaccination or could not be vaccinated (3).

It is surely imperative to address this critical topic; however, inaccurate interventions may backfire. In recent years, anti-vaccination movements have grown more structured and sturdier to criticism, relying on rhetoric and strongly refusing authority (4). Official communication is often met with disbelief, and lack of cohesion within the scientific community results in a failure to respond to the organized backlash of internet-based anti-vaccination movements (5).

Mandatory vaccination policies have also proven to be only partially effective: while increasing vaccination coverage, they are currently met with a significant degree of scepticism, sometimes evoking conspiracy sentiments (6, 7). People subjected to mandatory vaccination were found to fight it by pseudoscientific arguments (8), and even healthcare workers were observed to strongly oppose such measures (9). Therefore, different kind of interventions appear to be needed to fight hesitancy.

## Policy options

VH is currently recognized to be a multi-faceted phenomenon, rooted in both socioeconomic, cultural, and individual factors (1, 3). Communication regarding vaccination is therefore tricky: it should be simple enough to be understood by as many people as possible, yet with a complex structure. In fact, pro-vaccination messages should target different aspects of VH at once, account for the target audience’s diversity and use a technically correct but non-elitist language.

To date, however, several websites encouraging vaccination are often more difficult to understand for non-specialized readers than anti-vaccination platforms (10), and similar differences in readability are observed in various online settings (11). Classic communication based on dramatic narratives regarding the dangers of VH, despite still being widely used to sensitize the public about the importance of vaccination, has been proven to not be effective, while also stoking the fear of adverse events (12, 13).

When pondering and designing communication endeavors, policymakers should also take into consideration communication’s relationship with health literacy and vaccine literacy, specifically. In fact, according to a recent definition provided by Sørensen et al., (14) health literacy encompasses a variety of aspects, including “knowledge, motivation and competences to access, understand, appraise, and apply health information”. As far as vaccine-specific literacy is concerned, Lorini et al. (15) suggested that it is a “relational concept” related to one’s ability, motivation, and knowledge to seek, understand, appraise and apply information regarding vaccination in a larger conceptual workframe extending to themselves, their family, and their community. It is apparent that the “understanding” dimension of vaccine literacy can be at least partially impacted by communication’s quality.

## Actionable recommendations

First of all, medical communication should be accessible: relegating it to dedicated online databases makes it difficult to reach for the general audience. Providing additional sources of referenced information, both on digital and analog platforms, could help move medical notions closer to the public (16).

Secondly, VH often has a strong emotional component, and communication should take this factor into consideration (17). Addressing fear should be the top priority for all healthcare professionals, also considering that emotional wellbeing is part of the very concept of health (18). Additionally, the possibility of harm should be acknowledged and addressed properly to establish and maintain a stable relationship of communication and trust (13, 19, 20).

The role of frontline healthcare workers in promoting vaccination among their patients also represents a valuable asset. Various studies have highlighted how vaccine providers’ opinion was perceived as relevant by patients, contributing to orient their decision to accept the vaccine (21–23). By establishing a strong network of adequately trained healthcare workers at a community level, this positive influence might be expanded and result in an overall increase in vaccine acceptancy and vaccination coverage.

## Conclusion

Increasing people’s trust in healthcare professionals is a fundamental goal for modern healthcare systems. Patience is needed when talking to those who feel betrayed, abandoned, or even damaged by institutions. Most importantly, communication should be a profession: trained personnel should be responsible for spreading ideas the right way, making sure that everyone understands and no one is left behind.

Training of personnel should be adequately designed and directed in order to ensure the presence of competent frontline healthcare workers in all main healthcare settings. This aspect should be incorporated into governmental practice for uniformity’s sake, while its application should be curated by locally competent healthcare administration.

## Author contributions

AL: Conceptualization, Writing – original draft. PS: Methodology, Visualization, Writing – review & editing. ST: Conceptualization, Supervision, Writing – review & editing.
